# Structural insight into the recognition of amino-acylated initiator tRNA by eIF5B in the 80S initiation complex

**DOI:** 10.1186/s12900-014-0020-2

**Published:** 2014-09-17

**Authors:** Bernhard Kuhle, Ralf Ficner

**Affiliations:** 1 Abteilung für Molekulare Strukturbiologie, Institut für Mikrobiologie und Genetik, Göttinger Zentrum für Molekulare Biowissenschaften, Georg-August-Universität Göttingen, Göttingen, D-37077, Germany

**Keywords:** Ribosome, Translation initiation, Subunit joining, Initiator tRNA, eIF5B/IF2, Structure, Protein evolution

## Abstract

**Background:**

From bacteria to eukarya, the specific recognition of the amino-acylated initiator tRNA by the universally conserved translational GTPase eIF5B/IF2 is one of the most central interactions in the process of translation initiation. However, the molecular details, particularly also in the context of ribosomal initiation complexes, are only partially understood.

**Results:**

A reinterpretation of the 6.6 Å resolution cryo-electron microscopy (cryo-EM) structure of the eukaryal 80S initiation complex using the recently published crystal structure of eIF5B reveals that domain IV of eIF5B forms extensive interaction interfaces with the Met-tRNA_i_, which, in contrast to the previous model, directly involve the methionylated 3′ CCA-end of the acceptor stem. These contacts are mediated by a conserved surface area, which is homologous to the surface areas mediating the interactions between IF2 and fMet-tRNA^fMet^ as well as between domain II of EF-Tu and amino-acylated elongator tRNAs.

**Conclusions:**

The reported observations provide novel direct structural insight into the specific recognition of the methionylated acceptor stem by eIF5B domain IV and demonstrate its universality among eIF5B/IF2 orthologs in the three domains of life.

## 1 Background

The process of translation initiation results in the formation of an elongation-competent ribosome with the start codon of an mRNA in its P site, base paired to the amino-acylated initiator tRNA. In bacteria and eukarya this process follows significantly different mechanisms, highlighted by different numbers of auxiliary protein factors (initiation factors or IFs) that are employed by bacterial (three IFs) or eukaryal cells (at least 12 eIFs) for correct ribosome assembly [1]. Only two of these factors, a/eIF1A/IF1 and the translational GTPase a/eIF5B/IF2, are universally conserved in the three domains of life [2]. In bacteria, IF2 plays a critical role throughout the initiation pathway. In the early stages, IF2 binds to the 30S subunit in a GTP-dependent manner and stimulates the recruitment of the N-formylmethionylated initiator tRNA (fMet-tRNA^fMet^) to the P site of the 30S ribosomal subunit to form the 30S pre-initiation complex (pre-IC). Finally, IF2⋅GTP catalyzes the joining of the 50S ribosomal subunit to form the elongation-competent ribosome [3],[4]. Speed and accuracy of both processes depend of the specific recognition of the αNH-blocked methionine esterified to the 3′ CCA-end of tRNA^fMet^[5]-[8]. Biochemical studies showed that all determinants required for this interaction are located in domain IV of IF2, which consists of a six-stranded β barrel [9]-[12]. Domain IV of IF2 exhibits a marked structural homology to domain II of EF-Tu that, together with the G domain, forms the universally conserved structural core among translational GTPases [13] and in EF-Tu constitutes part of the binding pocket for the amino-acylated acceptor arm of elongator tRNAs [12],[14],[15]. Based on this observation it was suggested that IF2 domain IV and EF-Tu domain II use similar interfaces for their interactions with the tRNA [12]. This assumption is at least partially corroborated by mutational and NMR spectroscopy analyses [11]. Cryo-EM structures of bacterial 30S pre-ICs and 70S IC containing GTP/GDPNP-bound IF2 show how this interaction mutually stabilizes fMet-tRNA^fMet^ and IF2 in conformations that allow the efficient association of the 50S subunit [16],[17]. However, none of these structures were determined at sufficiently high resolution to give any detailed insight into the interaction that would allow a correlation with the biochemical data.

In contrast to bacterial IF2, the role of a/eIF5B in eukarya and archaea seems to be confined to the GTP-dependent promotion of subunit joining, the last step of the initiation process [18]-[20]. The recruitment of the charged initiator tRNA (Met-tRNA_i_) to the small ribosomal subunit is carried out by the heterotrimeric a/eIF2, a specialized EF-Tu paralog that has no counterpart in bacteria [21]. Accordingly, a/eIF5B⋅GTP binds to the small ribosomal subunit already containing the P site-bound Met-tRNA_i_, which invokes the question whether a/eIF5B still has to interact with Met-tRNA_i_ to promote joining of the large ribosomal subunit, and whether this interaction would involve a specific recognition of the methionylated acceptor end, similar to the recognition of the fMet-tRNA^fMet^ by IF2. Genetic, biochemical and structural studies point toward essentially the same mechanisms for eIF5B and IF2 catalyzed subunit joining [18],[19],[22]-[25]. Crystal structures of aIF5B and eIF5B revealed a six-stranded β barrel fold for domain IV, homologous to domain IV in IF2 [22],[24]. Indirect biochemical assays showed that a/eIF5B binds Met-tRNA_i_ in solution, however, with very low affinity and specificity for the methionyl moiety in case of eIF5B [22],[26]. NMR studies revealed a weak but specific interaction between methionine-ethyl ester (mimicking the ester bond between tRNA and the methionly moiety) and eIF5B domain IV in the area corresponding to the surface on IF2 that is affected by N-formylmethionine binding [27]. Finally, the recently determined 6.6 Å resolution cryo-EM structure of the yeast 80S IC (EM-Databank: EMD-2422) demonstrates that, like IF2 in the corresponding bacterial 70S complex [16], eIF5B and the P site-bound Met-tRNA_i_ stabilize each other in their subunit joining-competent conformations through the direct contact between domain IV and acceptor stem [25]. Surprisingly however, according to the structural model this contact does not involve the methionylated 3′ CCA-end of the tRNA [25]. Instead, the CCA-end points away from domain IV, placing the methionyl moiety ∼ 23 Å from the protein. Thus, this model is clearly at odds with the observations from biochemical studies [26],[27] and fails to explain why deacylation of the initiator tRNA results in the loss of its ability to stabilize eIF5B [25].

Here, we provide an analysis and reinterpretation for the cryo-EM density of the yeast 80S IC [25] for domain IV and its contact to the initiator tRNA. We show that the original structural model for this region, based on the fit of the archaeal aIF5B ortholog, is only partially consistent with the available density. Fitting of the recently determined structure of eIF5B domain IV from *C. thermophilum*, which shows a significantly higher degree of sequence similarity to the *S. cerevisiae* ortholog, allows a reinterpretation of the 6.6 Å resolution density. The resulting model demonstrates a direct contact between the methionylated CCA-end of the tRNA and a conserved surface area of domain IV that directly corresponds to the binding sites for the tRNA acceptor arm on domain IV of IF2 or domain II of EF-Tu [11],[12],[14]. Thus, we show that the high-quality cryo-EM density of the 80S complex not only provides the first direct structural indications for the EF-Tu-like interactions between eIF5B/IF2 domain IV and the initiator tRNA but also for their universality among a/eIF5B/IF2 orthologs in the three domains of life. Finally, we use our observations to propose a possible scenario for the evolution of the translational β barrel fold in eIF5B/IF2 and EF-Tu and its interactions with tRNAs.

## 2 Results and discussion

### 2.1 Model of eIF5B domain IV and the acceptor end of Met-tRNA_i_ in the 80S IC

**Figure 1:**
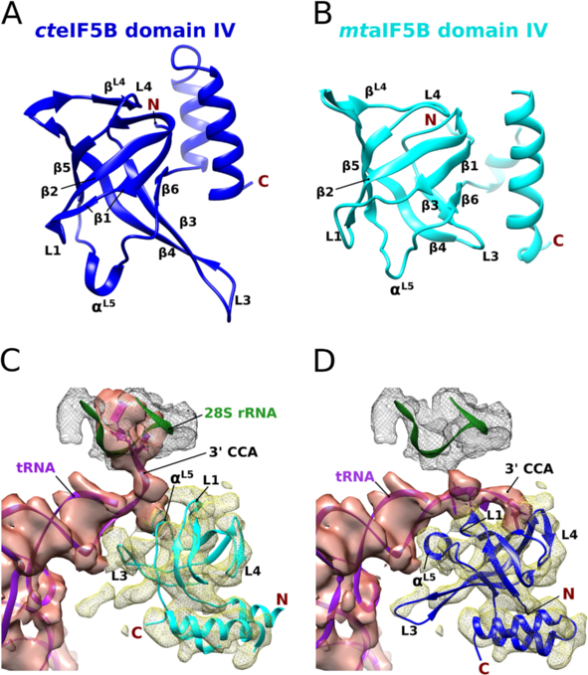
**Model for the interactions between eIF5B domain IV and Met-tRNA**_**i**_**in the 80S IC. A)** Crystal structure of *ct*eIF5B domain IV (PDB: 4N3G). The most marked differences to domain IV of aIF5B from the archaeon *M. thermoautotrophicum* (PDB: 1G7T) **(B)** are found in the lengths of the β3-β4 hairpin and loop L5 as well as in the arrangement of the two C-terminal α helices. **C)** Original model for the interactions between domain IV (cyan) and Met-tRNA_i_ (purple), fitted into the cryo-EM density of the 80S IC (EMD-2422) [25]. **D)** New model for the interactions between domain IV (blue) and Met-tRNA_i_ (purple), based on the rigid-body fitting of the crystal structure of domain IV from *ct*eIF5B. The 3′ CCA-end now forms a direct contact with the surface of domain IV, and α^L5^ occupies a position in the major groove of the acceptor stem.

Recently, we were able to solve two structures of eIF5B domain IV from the fungus *C. thermophilum*[24]. It consists of six antiparallel β strands (β1-β6) forming a closed β barrel that is followed by two α helices (Figure 1A). At its top and bottom, the β barrel is closed by an additional short β strand (β^L4^) and a one-turn α helix (α^L5^), respectively. Despite relatively low sequence similarity, it is structurally very similar to domain IV of aIF5B from the archaeon *M. thermoautotrophicum* (rmsd of 2.2 Å with ∼ 20% sequence identity and ∼ 30% similarity). However, in the *ct*eIF5B ortholog the β hairpin formed by β strands 3 and 4 and the loop following strand β5 (L5) contain 9 and 5 additional amino acids, respectively (Figure 1A/B). Further differences can be found in the organization of the two C-terminal α helices that are rotated by ∼ 25° with respect to the β barrel.

Compared to the archaeal ortholog, domain IV from *ct*eIF5B shows a relatively high sequence similarity to the yeast ortholog (19% sequence identity and 30% similarity for *mt*aIF5B compared to 49% identity and 65% similarity for *ct*eIF5B) including the β3-β4 hairpin, L5 and the two C-terminal α helices. Based on this observation, we assumed that the *ct*eIF5B structure allows a better fit to the recently determined cryo-EM density of the yeast 80S IC with initiator tRNA and eIF5B⋅GDPCP than obtained with the *mt*aIF5B structure [25] (Figure 1C). Rigid-body fitting of *ct*eIF5B domain IV (cross-correlation coefficient (CCC) of 73%) results in an improved correlation between structural model and density (Figure 1D): In contrast to the original fit of *mt*aIF5B [25], no clashes occur between the ribosomal RNA and *ct*eIF5B domain IV, as the loop between β strands 1 and 2 (L1) is moved away from C2284-U2286 and now lies next to the acceptor stem of the tRNA. Compared to the original model [25] the β barrel is rotated by ∼ 30°, causing the conserved helix α^L5^ at the bottom of the β barrel to displace the β3-β4 hairpin in the major groove of the initiator tRNA acceptor stem. In turn, the long, poorly conserved β3-β4 hairpin now occupies previously unexplained density close to the C-terminus of the last α helix and forms apparently no direct contacts to the tRNA (Figure 1D).

An interesting consequence of this reorganization of domain IV is the emergence of a well defined but unexplained density packed alongside the β barrel, directly opposite to the C-terminal α helices (Figure 1D). This density starts next to the very C-terminus of β strand 4 and the following loop (L4) and runs across strands β5, β2 and finally β1 where it directly leads into the continuous density of the phosphate backbone of the initiator tRNA at A73. Interestingly, this same position (A73) also marks the starting point for the distortion of the following 3′ CCA-end in the original model that is markedly different from its canonical conformation [25]. For the following reasons, it is unlikely that this original model gives the correct conformation for the tRNA acceptor arm in the 80S pre-IC: First, C75 and A76 clash extensively with the ribosomal RNA between G2615 and C2625 (Figure 1C). Second, the CCA-end is oriented away from eIF5B domain IV, resulting in a distance of ∼ 23 Å between the ribose of A76 (which carries the methionyl moiety) and the nearest parts of domain IV. This is clearly inconsistent with the observation that deacylation of the tRNA results in the loss of its contact to eIF5B in the 80S complex [25] and is at odds with the expected direct contact between the methionyl moiety and domain IV [19],[26],[27]. Remodeling of the 3′ CCA-end into the vacant density next to the β barrel of eIF5B avoids the clashes with the rRNA and, moreover, allows a direct recognition of the aminoacyl group by the protein by placing the 3′ end of the tRNA directly on top of the conserved Ala1056 of strand β5 (Figure 1D). It is important to note, that there is no alternative density present that could accommodate the entire CCA-end without causing a sterical conflict with the rRNA. Independent support for this new placement of the CCA-end is provided by the just recently published lower resolution (8–9 Å) cryo-EM model of the mammalian 80S pre-IC with eIF5B bound on HCV-IRES RNA, which suggests a direct contact between the acceptor end of the Met-tRNA_i_ and domain IV of eIF5B [28].

**Figure 2:**
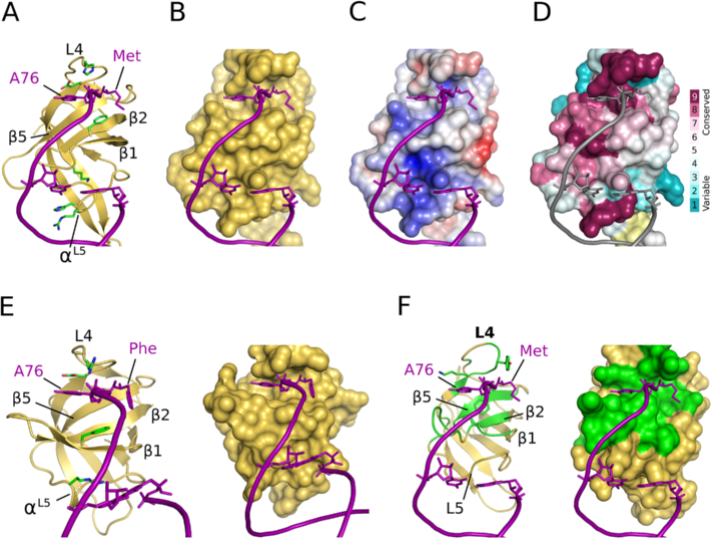
**The interaction interface between the acceptor stem of Met-tRNA**_**i**_**and domain IV of eIF5B.** Interactions between Met-tRNA_i_ (purple) and eIF5B domain IV **(A-D)**, Phe-tRNA^Phe^ and domain II of EF-Tu (PDB: 1TTT) **(E)** and fMet-tRNA^fMet^ and IF2 domain IV **(F)**. **A)** Domain IV of eIF5B in ribbon presentation (yellow) with residues potentially involved in interactions with the tRNA as green sticks. **B)** Surface presentation of domain IV, revealing the two well defined pockets below loop L4 that are also visible on the surfaces of EF-Tu domain II (E, right) and IF2 domain IV (F, right), and might accommodate A76 and the methionyl moiety of the 3′ CCA-end. **C)** Electrostatic surface potential of domain IV. **D)** Conservation of residues lying in the proposed interaction interface to the acceptor stem. **E)** Position of the acceptor stem of Phe-tRNA^Phe^ (purple) on the surface of domain II of EF-Tu (yellow; PDB: 1TTT). **F)** Model of domain IV of IF2 (PDB: 1D1N) and the initiator tRNA positioned as in **A)**. The green surfaces indicate residues of IF2 that were shown to interact with fMet-tRNA^fMet^[11],[12].

**Figure 3:**
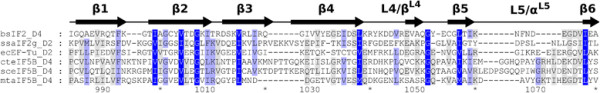
**Partial structure-based sequence alignment of the β barrel fold of domain IV (D4).** The aligned sequences are from *B. stearothermophilus* IF2, *M. thermoautotrophicum* aIF5B, *C. thermophilum* eIF5B and *S. cerevisiae* eIF5B with domain II (D2) from *E. coli* EF-Tu and *S. solfataricus* aIF2γ (GenBank: CAA27987, AAB84765, EGS21143, AAC04996, CAA40370, AAK40740, respectively). Highly conserved residues are highlighted in dark blue, conserved residues in light blue and similar residues in grey. Sequence numbering and secondary structure elements correspond to the *ct*eIF5B structure (PDB: 4N3G). As there is no structure of *sc*eIF5B domain IV available so far, its sequence was aligned directly with that of *ct*eIF5B.

As reported previously for *mt*aIF5B [22], the β barrel of eIF5B is structurally homologous to domain IV (C2 domain) of bacterial IF2 [12] and domain II of EF-Tu homologs, despite an overall low sequence similarity (Figures 2 and 3). Using site directed mutagenesis and NMR spectroscopy, it was shown that IF2 interacts with the αNH-formylmethionylated CCA-end of fMet-tRNA^fMet^ through a surface of domain IV that overlaps with that used by EF-Tu domain II to interact with the acceptor end of elongator tRNAs [11],[12],[14],[15]. The superposition of domain IV of *ct*eIF5B with domain IV of IF2 from *Bacillus stearothermophilus*[12] and domain II of EF-Tu from *Thermus aquaticus* in complex with Phe-tRNA^Phe^[14] reveals that these surface areas coincide perfectly with those occupied by the CCA-end in our cryo-EM density-based model (Figure 2). Consistently, a structure-based sequence alignment reveals the highest degree of sequence conservation between the eIF5B orthologs and EF-Tu domain II in those residues implicated in tRNA binding in IF2 and EF-Tu (Figure 3).

**Figure 4:**
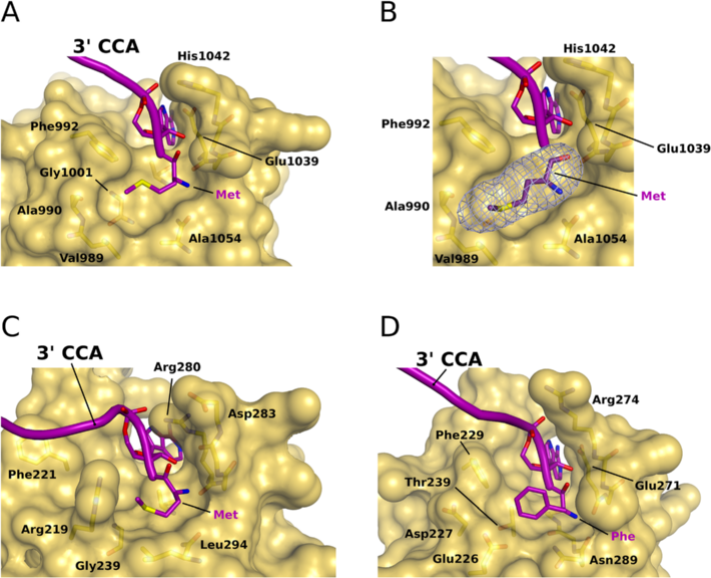
**Recognition of the methionylated 3′ CCA-end by eIF5B domain IV. A)** CCA arm, A76 and the methionly moiety of Met-tRNA_i_ modeled into the two surface pockets on eIF5B domain IV according to the cryo-EM density. Residues implicated in the interactions with the 3′ end are indicated. **B)** Simulated annealing f_o_-f_c_ omit map for the putative methionyl-binding pocket in eIF5B domain IV (blue mesh; contoured at 3σ). **C)** Met-tRNA_i_ bound to aIF2γ (PDB: 3V11). **D)** Phe-tRNA^Phe^ bound to EF-Tu (PDB: 1TTT).

The analysis of the surface area in *ct*eIF5B reveals two pockets next to the modeled 3′ end of the tRNA (Figures 2 and 4). The first formed by Val999, Gly1037, Glu1039 and the aliphatic part of Lys1058, corresponding to the EF-Tu residues Val237, Gly269, Glu271 and Leu289, respectively, which accommodate the base of A76 in Phe-tRNA^Phe^[14]. A similar pocket is found on the surface of IF2, whose residues are directly affected by fMet-tRNA^fMet^ binding [11],[12]. The second pocket is separated from the first by the methyl group of Ala1056 (corresponding to the conserved Gly287 in EF-Tu and Gly715 in *bs*IF2) and is formed on the one side by the hydrophobic Val989, Ala990, Phe992, Gly1001 and Ala1054 and on the other by the peptide backbone of Glu1039 to His1042. The position of this pocket corresponds to the localization of the aminoacyl groups in ternary complexes of EF-Tu, and residues of this area were found to interact specifically with N-formylmethionine in IF2 [11] and methionine-ethyl ester in eIF5B [27]. Consistently, this second pocket is compatible with the binding of a methionyl moiety in size as well as electrostatic surface properties (Figures 2 and 4). Notably, in both available crystal structures of *ct*eIF5B domain IV, this pocket is occupied by a large additional electron density. Due to the absence of alternatives in the crystallization condition (100 mM MES, 12% PEG 20000, 10 mM Na-lactate; ethylene glycol was used for cryo protection), this density was originally assigned to a lactate molecule [24]. However, refinement with the lactate molecule still results in positive difference electron density. A simulated annealing omit map for this area gives a density too large for a lactate (Figure 4B). Thus, the density would be compatible with the size of a methionine or other similarly large amino-acids whose α-carboxylate and α-amino groups form hydrogen bonds to the amide proton of Asp1041 and the main chain CO of Ala1054, respectively, corresponding to His273 and Asn285 that form similar contacts to the aminoacyl group in ternary complexes of EF-Tu [14],[29] (Figure 4D). However, the resolution of the structures (2.75 and 3.02 Å) necessarily does not allow an unambiguous assignment of the densities to a certain ligand, and a possible origin for a putative amino acid in this position remains elusive, as the weak binding between IF2 and fMet or eIF5B and methionine-ethyl ester [11],[27] makes a co-purification unlikely. The critical point, however, is the observation that the described pocket is evidently suited to accommodate organic molecules of a size similar to that of methionine and could thus accommodate the methionyl moiety of the Met-tRNA_i_ in a way analogous to domains II in EF-Tu and aIF2γ (Figure 4).

In ternary complexes of EF-Tu, the binding site for the 3′ CCA-end on domain II is complemented by the conserved Phe229 in strand β1 that stacks against C4 and C5 of the A76 ribose and by Arg274 (sometimes Gln or Lys) in the flexible loop following strand β4 that interacts with the phosphate of A76 [14],[29] (Figure 4C). The density assigned to the CCA-end of the Met-tRNA_i_ suggests similar interactions for the conserved Phe992 (in few cases Tyr or Ile) and His1042 in *ct*eIF5B (Figure 4A). According to the model, the rest of the acceptor stem of the tRNA adopts a slightly different orientation relative to the β barrel than observed for aa-tRNA bound to EF-Tu. In good agreement with the predictions made for IF2 [11], C75 and C74 seem to be rotated ∼ 20° toward the L1 loop (Figure 2). Interestingly, the orientation of the β barrel would allow several positively charged residues to interact directly with the acceptor stem. Lys994 in the L1 loop could contact the initiator tRNA specific A1:U72 base pair. The conserved Arg1070 and His1071 in helix α^L5^, positioned in a well defined density in the cryo-EM map (Figure 1D), are within contact distance to the phosphate backbone at G68 and C69 in the major groove. Notably, EF-Tu domain II contains a corresponding short helix α^L5^ in which the conserved Arg295 as well forms a contact to the acceptor arm of the bound tRNA [14],[29] (Figure 2E). Based on the comparison with EF-Tu it was previously assumed that a similar contact might be formed between Lys725 and Glu726 of *bs*IF2 and fMet-tRNA^fMet^. However, such an interaction could not be observed by NMR spectroscopy in solution [11]. It is therefore conceivable that these interactions are formed only in the context of the ribosomal pre-IC, where the tRNA is stabilized in a specific orientation relative to domain IV of eIF5B/IF2.

**Figure 5:**
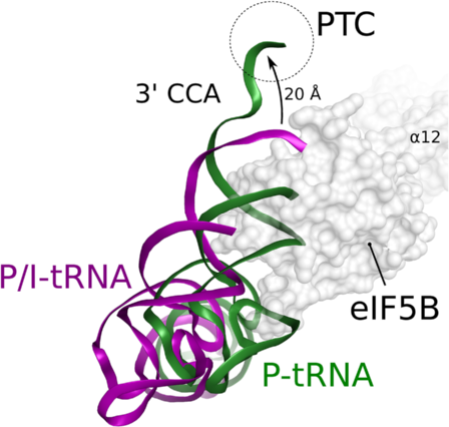
**Conformational rearrangement of Met-tRNA**_**i**_**on the initiation complex.** eIF5B stabilizes the initiator tRNA (purple) in a non-canonical P/I conformation [25] with the 3′ CCA-end outside of the PTC. Upon GTP hydrolysis in eIF5B and the release of the 3′ CCA-end from its contacts to domain IV, the initiator tRNA rearranges into the canonical P site conformation, involving a 20 Å repositioning of the 3′ end into the PTC.

Biological relevance of this domain IV-tRNA interaction lies in the mutual stabilization of initiator tRNA and eIF5B in conformations that allow efficient recruitment of the large ribosomal subunit and insertion of the acceptor arm into the peptidyl-transferase center (PTC). In the 80S complex domain IV stabilizes the tRNA in a non-canonical P/I orientation [25] that according to our model places the 3′ end ∼ 20 Å from the PTC without inducing a major distortion of the CCA-end from its canonical conformation (Figure 5). The following GTP hydrolysis and dissociation of eIF5B would thus allow the acceptor stem to rotate into the PTC while the overall tRNA relaxes into its canonical P site conformation. Through the specificity of domain IV for the methionylated acceptor arm, which might be more pronounced in the context of the preassembled 40S⋅Met-tRNA_i_ complex than in solution [26], this interaction would mark a final checkpoint in the initiation process that allows subunit joining only on correctly assembled 48S pre-ICs with a charged initiator tRNA bound in the P site [24],[30].

### 2.2 Implications for the evolution of the translational β barrel fold

**Figure 6:**
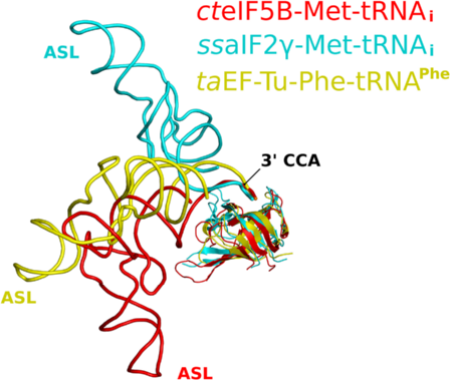
**Comparison of the interactions between eIF5B, EF-Tu and aIF2γ with tRNA.** Despite the same polarity and similar interfaces for the interactions between the translational β barrel fold and the single-stranded 3′ CCA-end, the tRNAs adopt significantly different overall orientations relative to the protein in ribosome-bound eIF5B (red) or the ternary complexes of *S. solfataricus* aIF2 (cyan; PDB: 3V11) and *T. aquaticus* EF-Tu (yellow; PDB: 1TTT). ASL is anticodon stem loop.

As reported previously, domain IV of IF2 and the structurally homologous domain II of EF-Tu use similar surface areas to interact with amino-acylated tRNAs [11],[12] (Figure 2E/F). The structure of the ternary complex of aIF2 shows the same interface for the interactions between domain II of aIF2γ (a paralog of EF-Tu) and the Met-tRNA_i_[31] (Figure 4C). Our observations provide structural evidence that this also applies to domain IV of eIF5B. This common binding interface for the 3′ CCA-end on the translational β barrel fold is centered on β strands 1, 2 and 5 and framed by the flexible loops L1 and L4 (Figures 2 and 4). In all cases additional interactions are made by the short capping α-helix that provides positively charged residues for contacts to the phosphate backbone of the acceptor stem, while at the same time allowing substantially different overall orientations of the tRNA relative to the β barrel, irrespective of an identical polarity of the bound CCA-end (Figure 6). Despite the low average sequence identity over the various β barrel folds (Figure 3), the significant structural and functional parallels in their interactions with amino-acylated tRNAs clearly point toward a common evolutionary origin. As eIF5B/IF2 and EF-Tu are both universally conserved in the three domains of life, their divergence and thus the origin for their respective tRNA-binding domains most likely lies long before the onset of speciation; this raises the interesting question of potential homologies to other ancient RNA-binding protein folds.

**Figure 7:**
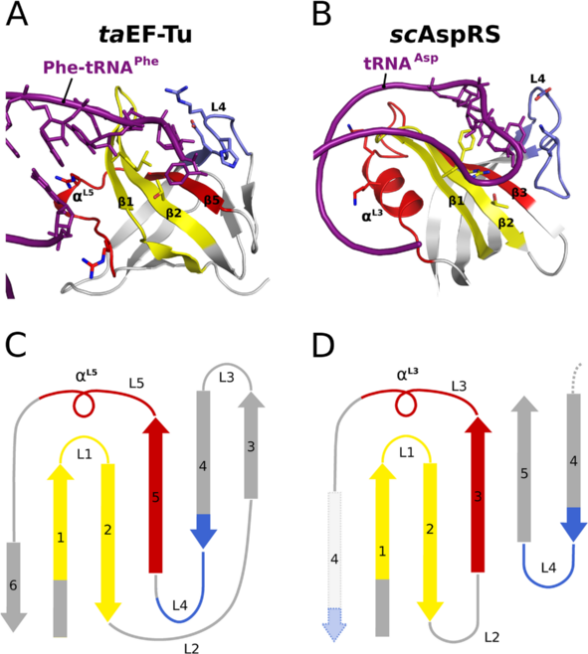
**Translational β barrel fold and OB-fold share the same fold related ligand-binding interface. A** and **C)** The interaction interface between the 3′ CCA-end of the tRNA and the translational β barrel (here Phe-tRNA^Phe^ (purple) bound to *T. aquaticus* EF-Tu domain II (PDB: 1TTT)) centers on β strands 1, 2 and 5 and is augmented by loops L1, L4 and L5, containing helix α^L5^. **B** and **D)** A related ligand-binding interface is found in single-stranded nucleic acid binding OB-folds (here the anticodon binding domain of the aspartyl-tRNA synthetase (PDB: 1ASZ) from *S. cerevisiae*[35]). Similar to the interactions observed for EF-Tu and despite a different topology, the bases of the anticodon stem loop (purple) point toward the surface of the β barrel, centered on β strands 1, 2 and 3, while the flexible loops L1, L4 and L3 with α^L3^ form additional contacts to the phosphate backbone.

A central question for the problem of cellular evolution is the appearance of the basic protein-folding types and of domains as functional building blocks for proteins. Folded proteins adopt only a limited number of folding structures; however, whether these folds emerged by divergent evolution from a single ancestor or independently by convergent evolution from different lineages is unclear. In this context, it is interesting that the characteristic features of tRNA binding by the translational β barrel fold show significant parallels to those between OB-fold domains and single-stranded nucleic acids (Figure 7). The OB-fold is a five-stranded mixed β barrel, capped on one end by an α-helix [32]. Most known OB-fold domains are involved in interactions with single-stranded RNA or DNA [33]. Despite very low sequence similarity among its members, the OB-fold superfamily is thought to be an ancient domain structure that derived by divergent evolution from a common ancestral protein – an assumption that is based on the common features of their fold-related ligand-binding interface [33],[34]. Despite a different overall topology of the OB-fold (Figure 7C/D) and a different classification in the SCOP (Structural Classification of Proteins) database, this interface, composed of β1-L1-β2-β3-L3/α^L3^-β4-L4 (Figure 7B), shows an intriguing structural and functional correspondence to the identically arranged but differently connected building blocks of β1-L1-β2 and β4-L4-β5-L5/α^L5^-β6 in the translational β barrel fold that are responsible for its interactions with tRNA (Figure 7A).

These similarities might merely be a functional analogy between both protein families that arose by convergent evolution from two distinct starting points. However, by the argument of a common descent based on a fold-related ligand-binding interface, the evident similarities might as well be indicative of a common evolutionary origin for the two equally ancient protein folds. For this hypothesis, two previously proposed theories are of particular interest: i) The emergence of domain folds by polyphyletic evolution from self-assembling short peptide ancestors, whose remnants (in sequence, structure or function) still exist in extant proteins [36]; and ii) the theory of a chemoautotrophic origin of life on volcanic iron-sulfur surfaces, according to which protein domains emerged from functional peptides that used metal ions as folding determinants or formed surface-bonded β-sheets that finally detached from the stabilizing surfaces (e.g. to form β-barrel domains) in the course of progressing cellularization [37]-[39]. In both theories, the transition from the peptide- to the independently folding protein-domain proceeds concomitant to the refinement of the genetic machinery that allows the synthesis of increasingly long polypeptides with sufficiently high fidelity [36],[38],[39].

In light of these hypotheses, we suggest a possible common polyphyletic origin of both fold-related RNA binding interfaces discussed above. At the earliest stages of cellular evolution, when the fidelity of the primordial translation apparatus allowed the synthesis only of short peptides, nucleic acid-peptide interactions most likely played an essential role, particularly for the genetic machinery. In this context, it would be conceivable that the common ligand-binding interface in the ancient lineages of OB-fold proteins and translational GTPases has arisen as an ancient structural entity formed by individually synthesized peptides, associating with single-stranded nucleic acids as folding determinants, similar to metallo-peptides as precursors for metallo-proteins [37]-[39]; during the gradual replacement of peptides by their fusion into independently folding proteins, this would ensure the conservation of the nucleic acid-binding interface, while at the same time allowing a substantially different connectivity of the individual building blocks in the emerging protein families.

## 3 Conclusions

In this study, we used the recently reported medium resolution cryo-EM density of the yeast 80S IC [25] and high resolution crystal structures of eIF5B from *C. thermophilum* to propose a new model for the interactions between eIF5B domain IV and the Met-tRNA_i_ in the context of the ribosome (Figure 1). According to this model, domain IV forms direct interactions with the phosphate backbone in the major groove of the acceptor arm, the initiator tRNA specific A1:U72 base pair and – most importantly – with the methionylated 3′ CCA-end. The relevance of these findings lies in the novel insight into the specific recognition of the amino-acylated initiator tRNA by eIF5B/IF2 in the context of pre-initiation complexes, which, as a final checkpoint for ribosomal subunit joining, is one of the central interactions in the process translation initiation. Finally, the identified binding interface between eIF5B and Met-tRNA_i_ directly corresponds to that reported earlier for the interaction between the homologous domains in IF2, EF-Tu and aIF2γ with their respective tRNA ligands [12],[14] and exhibits a striking structural and functional similarity to the fold-related ligand-binding interface of OB-fold domains, possibly reflecting a common evolutionary origin of the two ancient domain folds.

## 4 Methods

### 4.1 Model building

Rigid-body fitting of *ct*eIF5B domain IV (residues 382–1116; PDB codes 4N3N and 4N3G) was performed using UCSF Chimera [40]. Despite entirely different sets of crystal contacts for domain IV in the two X-ray structures they are very similar to each other (rmsd of 0.34), indicating a high degree of structural rigidity. Thus, although domain IV might undergo minor conformational changes upon interacting with ribosome and tRNA, particularly in the loop regions, we decided not to include any flexible fitting procedures to avoid overfitting of the model. Manual rebuilding of the acceptor stem of the Met-tRNA_i_ between bases G70 and A76 into the density next to domain IV was done in COOT [41]. Figures were prepared using UCSF Chimera [40] or Pymol (http://www.pymol.org).

We would like to mention here that in our hands the isolated domain IV of the *mt*aIF5B crystal structure (PDB: 1G7R) is fitted to the cryo-EM density in the same way as the *ct*eIF5B structure (with a CCC of 67%), supporting the newly proposed fit shown in Figure 1D. However, the *mt*aIF5B domain IV of the cryo-EM based model (PDB: 4BYX) is fitted as presented in [25] with a CCC of 78.7% (Figure 1C), most likely as the result of a combination of rigid-body and flexible fitting procedures [25], which gave rise to rmsds of 5.8 Å and 7.5 Å (over 106 C_α_ atoms) relative to the crystal structures of *mt*eIF5B and *ct*eIF5B, respectively, while the two crystal structures themselves differ only by an rmsd of 2.2 Å. Thus, the higher CCC for the cryo-EM-based model is most likely due to its distortion from the original rigid structure of *mt*aIF5B and is therefore not comparable to the CCC values obtained for our rigid-body fit.

### 4.2 Sequence alignments

Multiple sequence alignments were done using the iterative alignment program MUSCLE [42]. Structural sequence alignments were done using the DALI server [43].

## Abbreviations

IF: Initiation factor

eIF: Eukaryal initiation factor

aIF: Archaeal initiation factor

cryo-EM: Cryo-electron microscopy

pre-IC: Pre-initiation complex

EMD: EM-Databank

EF-Tu: Elongation factor-Tu

*ct*: *Chaetomium thermophilum*

*mt*: *Methanococcus thermoautotrophicum*

*sc*: *Saccharomyces cerevisiae*

*bs*: *Bacillus stearothermophilus*

PDB: Protein data bank

CCC: Cross-correlation coefficient

SCOP: Structural Classification of Proteins

OB-fold: Oligo-nucleotide/oligo-saccharide binding fold

rmsd: Root mean square deviation

PTC: Peptidyl-transferase center

ASL: Anticodon stem loop

## Competing interests

The authors declare that they have no competing interests.

## Authors’ contributions

BK designed the study, analyzed structures and cryo-EM density, performed the structural modeling and sequence alignments, analyzed and interpreted the data and wrote the manuscript. RF analyzed the data and helped to draft the manuscript. Both authors read and approved the final manuscript.
